# Observation on the Clinical Efficacy of Traditional Chinese Medicine Non-Drug Therapy in the Treatment of Insomnia: A Systematic Review and Meta-Analysis Based on Computer Artificial Intelligence System

**DOI:** 10.1155/2022/1081713

**Published:** 2022-10-11

**Authors:** Jingqing Zhuang, Jian Wu, Liang Fan, Chongnan Liang

**Affiliations:** ^1^Department of Nursing, Haikou Hospital of Traditional Chinese Medicine, Haikou, Hainan 570216, China; ^2^Department of Massage, Haikou Hospital of Traditional Chinese Medicine, Haikou, Hainan 570216, China; ^3^Department of Quality Control, Haikou Hospital of Traditional Chinese Medicine, Haikou, Hainan 570216, China

## Abstract

**Objective:**

Insomnia is a common and frequently occurring disease affecting the health of the population, which can seriously affect the work and life of patients. Drug treatment of insomnia has a rapid onset of action but has a large adverse reaction incidence rate. Traditional external treatment of traditional Chinese medicine (TCM) belongs to a type of non-drug therapy, the treatment of insomnia has a long history, but the methods of non-drug treatment of TCM are diverse, and the efficacy is also different. This study investigated the efficacy of TCM non-drug therapy in the treatment of insomnia by means of literature search and meta-analysis.

**Methods:**

We searched Embase, Pubmed, OVid, WOS, CNKI, and CBM for randomized controlled trials (RCTs) on the use of TCM as a non-drug treatment for primary insomnia. After doing a literature search according to the inclusion and exclusion criteria, we used Cochrane rob v2.0 to assess the potential for bias in the studies that were included, and we did a combined analysis and assessment of the effectiveness of the therapy.

**Results:**

16 articles were included in this study for quantitative analysis, and a total of 1285 patients participated in the study, including 643 patients in the intervention group and 642 patients in the control group. Meta-analysis showed that non-drug therapy of TCM could improve the treatment response rate of insomnia patients [*OR* = 6.88, 95%CI (4.40,10.74), *Z* = 8.48, *P* < 0.0001], reduce post-treatment *PSQI* total score [*MD* = −3.42, 95%CI (−4.62, −2.22), *Z* = −5.60, *P* < 0.0001], and improved patient anxiety [*SMD* = −1.25, 95%CI (−2.13, −0.37), *Z* = −2.78, *P*=0.01] and degree of depression [*SMD* = −1.53, 95%CI (−2.84, −0.21), *Z* = −2.28, *P*=0.02]. The heterogeneity survey showed that treatment time was one of the sources of heterogeneity. Meta-regression analysis revealed that publication year, patient age, sample size, and intervention characteristics were not specific factors affecting the combined results. *Discussion*. TCM non-drug therapy (acupuncture, moxibustion, massage, and auricular point pressing beans) can significantly improve the *PSQI* score of patients after treatment and improve the degree of anxiety and depression of patients, with significant effect, which is worthy of clinical promotion.

## 1. Introduction

Insomnia is characterized by difficulty falling asleep, easy awakening, and early awakening; it is a common and frequently occurring disease in clinical practice. According to epidemiological surveys, about 25%–30% of adults meet the diagnostic criteria of insomnia [1]. Insomnia is especially likely to occur in the elderly, women, family history, life stress, anxiety, perfectionism, and psychological diseases, and its predisposing factors include physical, psychological, environmental, lifestyle, drugs, and other factors [2]. The treatment of insomnia emphasizes comprehensive treatment, and identifying the cause is the key to the treatment of insomnia, while sleep hygiene education, psychotherapy, physical therapy, drug therapy, and traditional Chinese medicine (TCM) treatment are used to achieve the purpose of improving sleep quality, increasing effective sleep time, and restoring normal life and work [3]. Drug treatment for insomnia has a rapid onset of action but has a large adverse reaction incidence rate [4]. Related studies [5] have found that non-drug therapy for insomnia has the advantages of significant efficacy, high safety, and less adverse reactions, so the study of non-drug therapy for insomnia has become one of the hotter topics in recent years. Non-drug therapy mainly includes modern medical therapy and traditional external treatment of TCM. Modern medical therapy includes cognitive behavioral therapy, relaxation therapy, music therapy, and Morita therapy. Traditional external treatment of TCM includes acupuncture, auricular point pressing beans, massage, and other methods [6]. Insomnia belongs to the category of “sleeplessness” in TCM, and TCM has a long history of treating insomnia, but the methods of non-drug treatment in TCM are diverse and the efficacy varies [7]. There was not a significant change in PSQI score (Pittsburgh sleep quality index) between patients and controls after 4 weeks of acupuncture therapy, according to a paper written by Wang et al. [8]. The study was conducted on patients with insomnia. The PSQI score of insomnia patients following acupuncture therapy was substantially different from that of the control group, according to a controlled clinical investigation that was conducted by Yin et al. [9]. The study was conducted in a hospital setting. As a result of the aforementioned dispute, we decided to carry out this meta-analysis research in order to investigate whether or not quantitative meta-analysis is an effective way for resolving it.

## 2. Materials and Methods

### 2.1. Database and Search Strategy

We searched Embase, Pubmed, OVid, WOS, CNKI, and CBM databases until April 2022 for articles related to the treatment of primary insomnia with TCM using a keyword free search strategy containing: “*TCM*,” “*Tranditinal Chinese medcine*,” “*Acupuncture*,” “*Massage*,” “*Chronic insomnia*,” and “*Primary insomnia.*” Baseline conditions of participating study patients are shown in Table 1.

### 2.2. Inclusion Criteria

(1) Class of research: the works that we looked at were all randomized controlled trials (RCTs), and there were no language restrictions placed on them. (2) Study subjects: all study subjects were patients with continuous insomnia for more than one month and poor sleep quality, were over the age of 18, and were clinically diagnosed with insomnia [10]. (3) The intervention group: using TCM, it can be an intervention measure in acupuncture, moxibustion, decoction, Chinese patent medicine, and massage. (4) The control group: sham treatment or no intervention or general traditional western medicine treatment measures were taken. (5) The results of the study showed that the intervention group treatment results are effective. (6) Outcome indicators: it is possible to offer at least one outcome indicator, such as the response rate, the score on the Pittsburgh sleep quality index (PSQI), the recurrence rate, anxiety, depression, the incidence of adverse reactions, and comprehensive post-intervention data.

### 2.3. Exclusion Criteria

**Figure 1:**
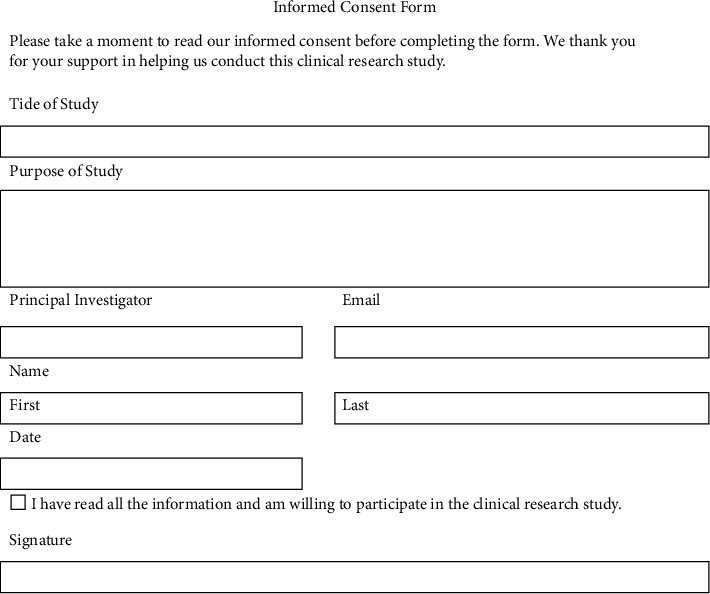
Informed consent form template.

Exclusion criteria include the following: (1) patients who suffer from non-primary insomnia such as those whose sleeplessness is brought on by conditions such as maintenance blood diseases, menopause in women, cancer-related issues, moderate to severe anxiety disorders, moderate to severe depression, convalescent strokes, Parkinson's disease, schizophrenia, and other medical conditions. The following types of research will not be considered: (2) literatures on combined Chinese and Western medicine treatment in interventions; (3) investigations, case analyses, and reviews of non-randomized controlled studies will not be considered; (4) studies with missing outcome indicators, unavailable data, or untransformable data will not be considered (Figure 1).

### 2.4. Screening of Literature

After literature retrieval, the repeated literatures were excluded by software. Two researchers read the title and abstract, screened according to the inclusion and exclusion criteria, and obtained the full text of the remaining literatures. If the original text could not be obtained from the Internet, the author of the original text was contacted, read the full text of the literatures, and further screened.

### 2.5. Interview Participants

Interview participants will be invited from 5 practices from each of the three trial recruitment centres. The practices will be selected to reflect a range of practice types (e.g., based on practice size or membership of a consortium). One practice nurse, one trial participant, and one practice manager or GP will be interviewed from each selected practice. We have added further information to clarify.

### 2.6. Data Extraction

Two researchers independently extracted literature data: literature author, publication year and month, grouping method, number of cases in each group, patient age, gender ratio, initial *PSQI* score, duration of insomnia, intervention measures, treatment time, follow-up time, and outcome indicators. After data extraction was completed by both researchers, each other's results were cross-checked and discrepancies were discussed and finalized.

### 2.7. Statistical Methods

(1) Effect sizes were reported as Odd Ratio (*OR*) with 95%CI for discrete variables; (2) effect sizes were reported using Mean Difference (*MD*) or Standard Mean Difference (*SMD*) with 95%CI for continuous variables; (3) comparisons were performed using forest plot descriptive statistics; (4) literature heterogeneity was analyzed using *I*^2^ analysis and *Q* test with *I*^2^>50% or *P* < 0.1 indicating heterogeneity of the results; (5) if there was no heterogeneity between the literature, it was calculated by Mantel-haeszel method; if there was heterogeneity between the literatures, it was calculated by Dersimonian-Laird method; (6) heterogeneity survey: subgroup analysis was used to investigate heterogeneity; (7) meta-regression analysis: meta-regression analysis was used to investigate factors that were significant for effect size; (8) sensitivity analysis: detect literature with the greatest impact on effect size; and (9) detect publication bias using Egger's test and present using funnel plot.

## 3. Results

### 3.1. Literature Screening Results

**Figure 2:**
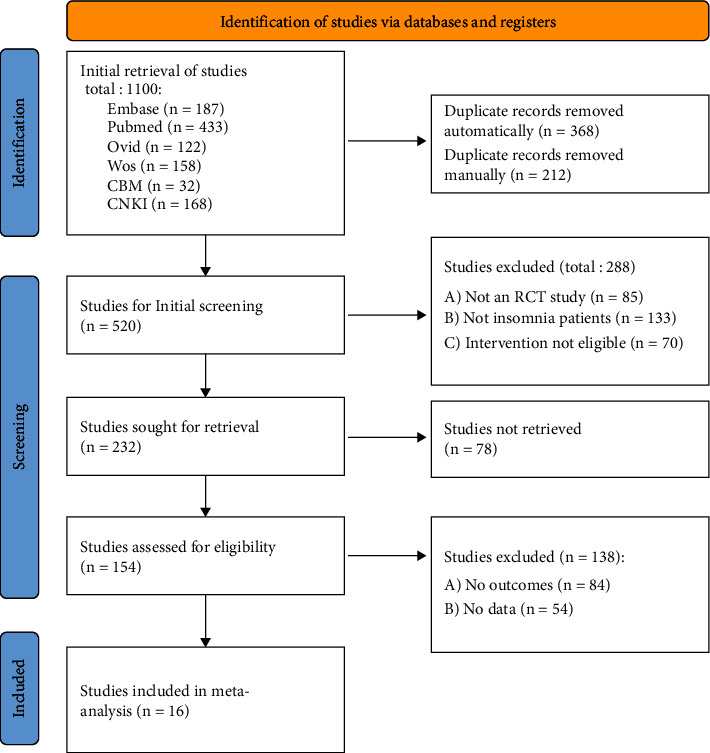
Literature selection flow chart.

The literature selection flow chart is shown in Figure 2, 1100 articles were initially retrieved, and after de-duplication and screening, 16 articles were finally included in the quantitative analysis, as shown in Table 2.

### 3.2. Literature Quality and Bias Evaluation

**Figure 3:**
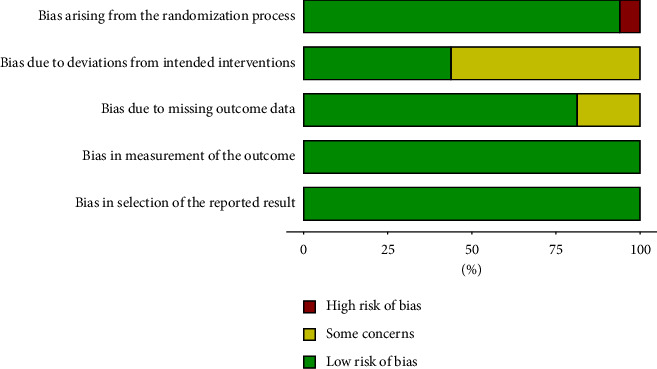
Bias analysis of randomized controlled intervention based on ROB 2.0.

In this study, the literature [22] grouped according to the order of admission and did not strictly use the random sequence process, which may have a large bias; all other literatures described the generation method of random sequence (using permuted block randomization method or computer random sequence generation method). Literatures [17–23, 25, 26] did not describe allocation concealment method and blinding method; others used sealed opaque envelopes to conceal numbers and implemented blinding method. All literatures recorded dropout cases in detail, without significant selective reporting bias and other biases. The overall quality was excellent, as shown in Figure 3.

### 3.3. Meta-Analysis Results

#### 3.3.1. Effective Rate of TCM Non-Drug Therapy Acting on Primary Insomnia

**Figure 4:**
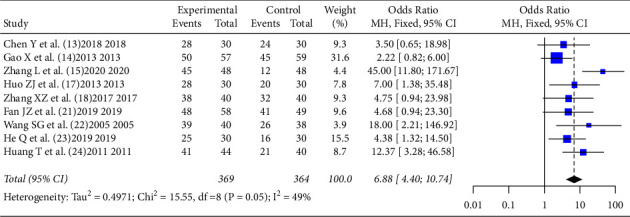
Effect of TCM non-drug therapy on the treatment efficiency of patients with primary insomnia.

In the literatures [13–15, 17, 18, 21–24], a total of 9 literatures reported the effective rate after intervention. There was no statistical heterogeneity between the literatures (*I*^2^ = 49%, *P*=0.05), using fixed effect mode, and meta-analysis showed that TCM non-drug therapy could improve the effective rate of insomnia patients [*OR* = 6.88, 95%CI (4.40,10.74), *Z* = 8.48, *P* < 0.0001], as shown in Figure 4.

#### 3.3.2. Effect of TCM Non-Drug Therapy on *PSQI* Index in Patients with Primary Insomnia

**Figure 5:**
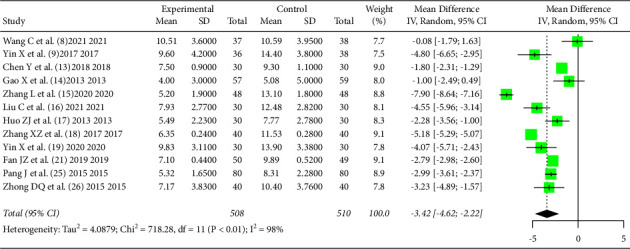
Effect of TCM non-drug therapy on *PSQI* score of patients with primary insomnia after treatment.

*PSQI* indicators after intervention were reported in all 12 literatures [8, 9, 13–19, 21, 25, 26], and there was statistical heterogeneity between the literature (*I*^2^ = 98%, *P* < 0.01), using random effect model, and meta-analysis statistics showed that TCM non-drug therapy could reduce the total *PSQI* score after treatment [*MD* = −3.42, 95%CI (−4.62, −2.22), *Z* = −5.60, *P* < 0.0001], as shown in Figure 5.

#### 3.3.3. Effect of TCM Non-Drug Therapy on Other Indicators in Patients with Primary Insomnia

3 articles [13, 14, 16] reported the recurrence rate after treatment, 4 articles reported the degree of anxiety and depression of patients after treatment, and 3 articles reported the incidence of adverse reactions after treatment, as shown in Table 3.

#### 3.3.4. Investigation of Sources of Heterogeneity

In the analysis of *PSQI* indicators, there was statistical heterogeneity between the literatures (*I*^2^ = 98%, *P* < 0.01). Subgroup analysis was performed for the literatures according to “intervention method,” “control group method,” and “intervention time.” The heterogeneity test between subgroups after grouping only “intervention time” showed *P* < 0.05, indicating that “intervention time” was one of the sources of heterogeneity in this study, as shown in Table 4.

#### 3.3.5. Meta-Regression Analysis

**Figure 6:**
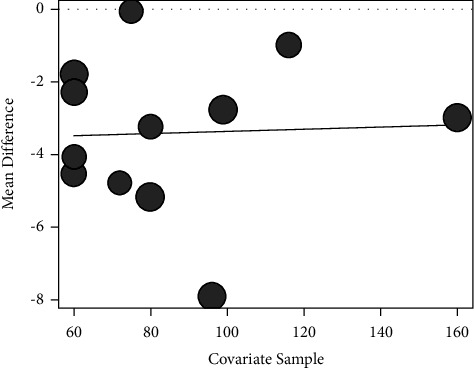
Meta-regression analysis of *PSQI* score outcome indicators: publication year factor.

In the analysis of *PSQI* index, we regressed pooled ES using “publication year of literature,” “study sample size,” “mean age,” and “intervention method + control group method + treatment time” and found that the *P* values of the effects of these four factors on *PSQI* index ES were 0.32, 0.90, 0.15, and 0.23. That means none of these factors could linearly affect the results of the meta-analysis (Figure 6).

#### 3.3.6. Sensitivity Analyses

**Figure 7:**
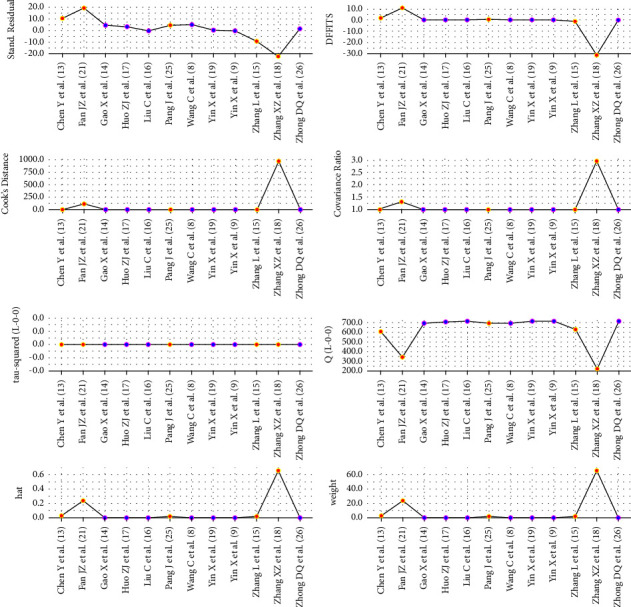
*PSQI* score effect size impact analysis.

Our impact analysis on the combined effect size of *PSQI* outcome indicators showed that the literature [18] was the most influential literature, and excluding these two articles, the combined effect size of *PSQI* outcome indicators remained statistically significant, showing good stability of the results (good sensitivity) as shown in Figure 7.

#### 3.3.7. Publication Bias Analysis

**Figure 8:**
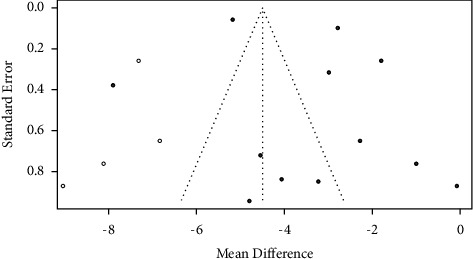
Funnel plot for *PSQI* outcome indicators.

For the pooled effect analysis of *PSQI* outcome indicators, publication bias was detected by Egger's test: *P* = 0.30, which did not indicate the existence of asymmetry in the funnel plot, which is shown in Figure 8.

## 4. Discussion

At present, the main means of insomnia treatment is drug therapy, sedative hypnotics represented by benzodiazepines are effective, but adverse reactions are obvious, long-term medication can lead to psychomotor impairment, memory impairment, drug addiction, aggravation of depression and rebound insomnia after withdrawal, and other negative effects. In addition, it is easy to produce adverse reactions such as fatigue, dizziness, and drowsiness [27, 28]. Physical therapy emerging in recent years has been able to improve sleep quality in patients to varying degrees with less adverse reactions, but it has not been clinically promoted because its mechanism of action is not clear [29]. TCM characteristic therapy has been gradually paid attention and widely used in the treatment of insomnia, especially the traditional external treatment of TCM such as acupuncture and massage developed on the basis of the theory of meridians and viscera in TCM, which has the advantages of good clinical efficacy and less adverse reactions [30].

In this study, 16 high-quality RCT studies with a total of 1285 participants were included, and the results showed that non-drug therapy of TCM (acupuncture, moxibustion, massage, and auricular point pressing beans) could significantly improve the *PSQI* score of patients after treatment, the effective rate was significantly higher than that of the control group, and the improvement of anxiety and depression was better than that of the control group. Acupuncture therapy is the most widely used, long-standing treatment and is clinically mainly based on dialectical acupoint selection but also using specific acupoints for treatment. The main points mainly take Baihui, Sishencong, Shenmen, Sanyinjiao, hypnosis, and so on. At the same time, a group of matching points were selected based on syndrome differentiation: if the liver stagnated fire, Taichong, Fengchi, Yanglingquan, and Zhimen points were taken; if the phlegm-heat internal disturbance, Fenglong, Houxi, Shenmai, Daling, and Lidui points were taken; if the heart and spleen were deficient, Neiguan, Zusanli, Xinshu, and Pishu points were taken; if the *yin* deficiency and fire flourishing, Taixi, Taichong, Xinshu, Shenshu, Zhaohai, Daling, and Fuliu points were taken [8, 9, 15–18]. Acupuncture can regulate viscera in the treatment of insomnia and has the advantages of safety and no side effects. Massage is also commonly used in the treatment of insomnia, manipulation mostly acts on the head of the human body, healers often massage with acupuncture, so that the effect is significantly and lasting. Massage therapy for insomnia is to use certain techniques to stimulate the fixed parts of the human body and play a role in stimulating meridians and *qi* and balancing *yin* and *yang*. In addition, it can dredge *qi* and blood, improve tissue oxygen supply, and regulate the relative balance of excitation and inhibition of the nervous system. Repeated stimulation of the head and face with gentle maneuvers can excite peripheral nerves, increase blood circulation in the head and face, improve brain tissue nutrition, and play a role in inhibiting the central nervous system, ultimately achieving tranquilizing purposes [24–26]. Moxibustion uses the heat of fire to give the patient warm stimulation of the human body, through the action of meridians, to achieve the effect of improving insomnia [13, 14]. Other methods such as auricular acupressure, acupoint injection, cupping, and fumigation are effective, but there is a lack of clinical controlled studies, so this study was not included.

Although all studies with subjects with non-primary insomnia (women with menstrual insomnia, tumor-induced insomnia, post-stroke insomnia) were strictly screened out in this study, significant heterogeneity between the literature was still observed when combining the analyses. In this study, subgroup analysis was performed according to different interventions, and it was found that although the interventions taken in the literature were different and the interventions in the control group were also different, the intervention methods were not the cause of heterogeneity, but the treatment time contributed the most to heterogeneity and was one of the sources of heterogeneity. We failed to find all the heterogeneity contributors, and statistically significant heterogeneity remained within the subgroups after passing through the subgroup groups, the source of which may be related to the characteristics of the different participants.

We tried to find factors affecting the combined results by meta regression analysis but did not find statistically significant factors. According to the impact analysis, the literature [18] contributed the most to the difference in the results, but excluding the literature [18] did not change the combined results of the meta-analysis, which indicated that this analysis was stable.

In this study, it was also found that there was no significant difference in the recurrence rate and incidence rate of adverse reactions between the observation group and the control group, but there were still too few literatures included for these two indicators.

In this study, the funnel plot showed that both sides were symmetrically distributed, suggesting that the publication bias was small (which was also confirmed by Egger's test). However, there are still some literatures with high risk of bias included in this meta-analysis, and there are still few literatures included in each intervention method. Therefore, RCT studies with larger sample size are still required to be further explored for the study on this topic.

A total of 1285 patients were included in 16 literatures in this meta-analysis. The results showed that TCM non-drug therapy (acupuncture, moxibustion, massage, and auricular point pressing) could significantly improve the *PSQI* score of patients after treatment, with an effective rate significantly higher than that of the control group, and the improvement of anxiety and depression was better than that of the control group. However, due to the small number of articles included in this study, randomized controlled studies with larger sample sizes remain to be explored in depth on this topic. On the other hand, the influence of commonly used anti infective drugs or anti-tumor drugs on the mental state of patients also deserves attention [11, 12, 31–33].

## Figures and Tables

**Table 1:** Baseline conditions of participating study patients. Basic characteristics of the informed consent form.

Factors	Number of patients (%)
Gender (%)	
Male	31 (88.5)
Female	4 (11.5)
Median age, years (range)	50 (34–72)
Median follow-up months	14
Median time interval between initial and reirradiation	51 (12–240)

**Table 2:** Basic characteristics, intervention measures, treatment time, and outcome indicators of included literatures.

Author and publication date	Number of cases	Number (E/C)	Age (years)	Intervention category	Intervention measures	Control intervention	Treatment time	Rehabilitation index
Wang et al. [8] 2021	75	37/38	57.9 ± 8.76	Acupuncture	Acupoints: HT 7 and KI 7	Sham treatment	3 weeks	②
Yin et al. [9] 2017	72	36/36	39.7 ± 12.9	Acupuncture	Acupoints: Baihui (GV20), Shenting (GV24), Yintang (GV29), Bilateral Anmian (EX-HN22), Bilateral Shenmen (HT7), Bilateral Sanyinjiao (SP6)	Sham treatment	4 weeks	②⑤⑥
Chen et al. [13] 2018	60	30/30	45.3 ± 11.2	Moxibustion	Pricking and penetrating moxibustion therapy	Conventional treatment	3 weeks	①②
Gao et al. [14] 2013	116	57/59	40 ± 13	Moxibustion	Moxibustion at Baihui (GV20) and Sishencong (EX-HN1)	Conventional treatment	3 weeks	①②
Zhang et al. [15] 2020	96	48/48	37.9 ± 14.1	Acupuncture	Acupoints: Anmian (EX-HN22), nNeiguan (PC6), Shenmen (HT7), Hegu (LI4), Zusanli (ST36), Zhaohai (KI6), Shenmai (BL62) and Taichong (LR3)	Sham treatment	4 weeks	①②③⑤⑥
Liu et al. [16] 2021	60	30/30	47.17 ± 14.08	Acupuncture	Acupoints: Baihui (GV20), Yintang (GV29), Shenmen (HT7, bilateral), and Sanyinjiao (SP6, bilateral)	Sham treatment	4 weeks	②⑤⑥
Huo et al. [17] 2013	60	30/30	46.00 ± 10.7	Acupuncture	Acupoints: Baihui (DU20), bilateral Zusanli (ST36), Neiguan (PC6), Shenmen (HT7), Sanyinjiao (SP6), Taichong (LR3), and Yongquan (KI1)	Conventional treatment	4 weeks	①②
Zhang et al. [18] 2017	80	40/40	37.45 ± 4.25	Acupuncture	Acupuncture and moxibustion points: Shenmen, Neiguan, Fengchi, Taichong, Xingjian, Zusanli, Taixi, and Sanyinjiao points	Sham treatment	8 weeks	①②
Yin et al. [19] 2020	60	30/30	47.3 ± 14.9	Electroacupuncture	Baihui (GV20), Shenting (GV24), Yintang (GV29), Bilateral Anmian (EX-HN22), Shenmen (HT7), Sanyinjiao (SP6), and Neiguan (PC6)	Sham treatment	8 weeks	②③⑤⑥
Xing et al. [20] 2020	63	31/32	54.45 ± 12.1	Electroacupuncture	Acupuncture points used were DU-20, EX-HN1, EX-HN22, SP-6, HT-7, PC-6, BL-62, and KI-6	Conventional treatment	4 weeks	⑤⑥
Fan et al. [21] 2019	99	50/49	68.52 ± 5.38	Acupuncture	Acupuncture: Acupuncture at Anmian point	Conventional treatment	4 weeks	①②③④
Wang et al. [22] 2005	78	40/38	NR	Acupuncture	Acupuncture and moxibustion points: the main points are Zhaohai and Shenmai. Acupoints: according to syndrome differentiation, liver stagnant fire type to take Neiguan, Xingjian, Ganshu; phlegm-heat internal disturbance type to take Shenmen, Neiguan, Gongsun, Fenglong; Yin deficiency and fire excess type to take Taixi, Xinshu, Shenshu; heart and spleen deficiency type to take Xinshu, Pishu, Zusanli, sanyinjiao; heart and gallbladder qi deficiency type to take Daling, Danshu, Ganshu, Yin Xie	Sham treatment	2 weeks	①
He et al. [23] 2019	60	30/30	41.4 ± 11.0	Acupuncture + auricular point pressing	Acupuncture points: Shanggen, Yintang, Anmian, Xingjian (bilateral), Taichong (double) side) Auricular point pressing beans: select Shenmen, sympathetic, subcortical, heart and liver as the main acupoints	Conventional treatment	4 weeks	①
Huang et al. [24] 2011	84	44/40	44.6 ± 12.5	Massage	Foot bath + plantar massage	Conventional treatment	2 weeks	①
Pang et al. [25] 2015	160	80/80	45.83 ± 9.02	Massage	Acupoints on head: Yangbai, Benshen, Head Lin Weeping, Zhengying, Chengling, Rugu, Tianchong, Bubai, Tip Yin, Fengchi	Conventional treatment	4 weeks	②
Zhong et al. [26] 2015	80	40/40	33.0 ± 4.1	Massage	Acupressure	Conventional treatment	3 weeks	②⑤

**Table 3:** Meta-analysis results of other outcome indicators.

Factors	Reported literature	Literature number	Analysis mode	*I* ^2^ C with *P* value	Effect size	Pooling value	*Z*, *P* value
Relapse rate	21, 23	2	Random effect mode	85.8% with 0.008	*OR*	0.04[0.00, 0.97]	−1.98, 0.05
Anxiety level	9, 15-16, 19-20, 26	6	Random effect mode	93.0% with 0.001	*SMD*	−1.25[−2.13, −0.37]	−2.78, 0.01
Degree of depression	9, 15-16, 19-20	5	Random effect mode	95.5% with 0.001	*SMD*	−1.53[−2.84, −0.21]	−2.28, 0.02
Incidence of adverse reactions	14–15, 21	3	Random effect mode	54.0% with 0.11	*OR*	0.47[0.11, 2.04]	−1.02, 0.31

**Table 4:** Subgroup analysis of *PSQI* indicators.

No.	Grouping method	Subgroup	Literature number	Heterogeneity		*P* value
				I^2^	*P*	
1	Intervention methods	Acupuncture	7	94.5%	<0.001	0.08
		Moxibustion	3	88.6%	0.05	
		Massage	2	0	0.99	
2	Control group method	Sham intervention	6	94.3%	<0.001	0.05
		Traditional treatment	6	74.2%	0.04	
3	Intervention time	3 weeks	4	61.0%	0.03	0.0001
		4 weeks	6	97.2%	<0.001	
		8 weeks	2	42.6%	—	

## Data Availability

The data used in this study are available from the author upon request.
